# Effects of Biochar Application on Soil Organic Carbon Composition and Enzyme Activity in Paddy Soil under Water-Saving Irrigation

**DOI:** 10.3390/ijerph17010333

**Published:** 2020-01-03

**Authors:** Shihong Yang, Xi Chen, Zewei Jiang, Jie Ding, Xiao Sun, Junzeng Xu

**Affiliations:** 1 State Key Laboratory of Hydrology-Water Resources and Hydraulic Engineering, Hohai University, Nanjing 210098, China; xjz481@hhu.edu.cn; 2 College of Agricultural Engineering, Hohai University, Nanjing 210098, China; sunrise@hhu.edu.cn (X.C.); 171302060019@hhu.edu.cn (Z.J.); hhudingjie@hhu.edu.cn (J.D.); sx1027@hhu.edu.cn (X.S.); 3 Cooperative Innovation Center for Water Safety & Hydro Science, Hohai University, Nanjing 210098, China

**Keywords:** paddy fields, straw biochar, water-saving irrigation, soil organic carbon, soil enzyme activity

## Abstract

Rice water-saving irrigation technology can remarkably reduce irrigation water input and maintain high yield; however, this technology can also accelerate the decomposition of soil organic matter in paddy fields. The spatial and temporal distributions of soil organic carbon (SOC), water-soluble organic carbon (WSOC), and soil microbial biomass carbon (SMBC) under different water-carbon regulation scenarios were analyzed on the basis of field experiments in the Taihu Lake region in China to explore the effects of biochar application on SOC and its components in water-saving irrigation paddy fields. The response of soil catalase (CAT) and invertase (INV) to biochar application in water-saving irrigated rice fields was clarified. The results showed that water-saving irrigation reduced the SOC content by 5.7% to 13.3% but increased WSOC and SMBC contents by 13.8% to 26.1% and 0.9% to 11.1%, respectively, as compared with flooding irrigation. Nonflooding management promoted the oxidative decomposition of soil organic matter. Two years after straw biochar was added, paddy soil SOC content under water-saving irrigation was increased by 4.0% to 26.7%. The WSOC and SMBC contents were also increased by 4.0% to 52.4% and 7.0% to 40.8%, respectively. The high straw biochar addition rate exhibited great impact on SOC. Remarkable correlations among SOC, WSOC, and SMBC were observed, indicating that the addition of straw biochar improved soil labile C, such as WSOC and SMBC, which promoted SOC transformation and stability in paddy soil under water-saving irrigation. Soil CAT and INV were related to SOC conversion. In conclusion, the combination of water-saving irrigation and straw biochar addition was beneficial to the improvement of soil properties and fertility of paddy fields.

## 1. Introduction

Carbon cycle is closely related to global energy balance and ecosystem productivity [1], and the soil carbon pool is the largest in terrestrial ecosystems [2]. Approximately 1500 to 2000 Pg (1 Pg = 10^15^ g) of carbon is stored in soils in the form of organic carbon [3,4], accounting for more than half of the soil carbon pool worldwide; soil organic carbon (SOC) that exhibits active exchange with atmospheric components accounts for approximately two-thirds of the terrestrial ecosystem carbon [5]. The organic carbon in the soil is a carbon sink and source, and its small changes greatly alleviate or accelerate the concentration of atmospheric CO_2_, thereby changing the global carbon cycle [6]. Soil carbon is the balance between carbon input into and loss from the soil. In the wake of global warming, especially in recent decades, the existing dynamic equilibrium between soil and atmospheric pools has been shifted by climate and human intervention, and a large amount of SOC has been oxidized and released into the atmosphere in the form of CO_2_ due to the long-term and large-scale global farming, thereby resulting in high emissions of greenhouse gases [7]. Land use change caused by human activities, including farming, is a main driver to terrestrial ecosystem carbon storage. Approximately 10% of the world’s land is used for agricultural production [8]. Farmland soil carbon pools are more active and susceptible to human activities than other soils, but they can also simultaneously self-adjust in a short time [9]. In recent years, researchers in China and abroad have paid considerable attention to the role of farmland soil in carbon fixation. In regions with high agronomical pressure, carbon management has a remarkable influence on crop productivity and yield [10]. Farmland SOC pool, an important part of the soil carbon pool, can affect farmland productivity by influencing soil quality and global carbon cycle by affecting atmospheric CO_2_ concentration [11]. Therefore, SOC and its components in farmland must be investigated and analyzed to achieve global ecosystem carbon balance and sustainable utilization of farmland soil.

Paddy field area accounts for 23% of the total cultivated land in China and 20% of the total rice planting area in the world [12]. Winter warming, a result of greenhouse effect, has been observed recently in southern China, where 90% of paddy soils in China are distributed [13]. Paddy soil exhibits a high level of organic carbon content, an evident trend of carbon sequestration, and a great potential for carbon fixation, which is an important part of the construction of soil carbon sequestration capacity to reduce greenhouse gas emission. In recent years, most studies of SOC composition in paddy fields focused on the impact of land use mode, farming practices, or fertilization on its content but less on water management and biochar application [14,15,16]. Related research only focused on the effects of single factors, such as irrigation or biochar, on SOC [13,17]. Previous studies have shown that frequent wet–dry alternation in water-saving irrigation paddy fields promotes soil respiration [18] and remarkably stimulates CO_2_ release from soils [19,20]. The application of water-saving irrigation reduces the SOC content in paddy fields. Water-saving irrigation should be combined with farmland carbon management measures, such as straw returning, organic fertilizer application, and biochar application, to realize the sustainable utilization of water and carbon resources in paddy fields.

Biochar is a by-product of the residual carbonization of biomass organic carbon after being separated from combustible gases under anaerobic or hypoxic conditions and high-temperature pyrolysis. This by-product presents a high degree of aromatic ring molecular structure and porous characteristics [21] and higher thermal stability and stronger adsorbability than general organic materials. Biochar application is considered a potential effective measure to increase carbon retention in soils and mitigate the effects of greenhouse gas [22,23]. Many studies have shown that the application of biochar can rapidly enhance soil carbon pool [24]. Applying biochar has beneficial effects on soil active organic carbon components, such as an increase in crop biomass and input of fresh organic carbon [25], improvement in soil structure, promotion of soil aggregate formation [26], and provision of an ideal habitat for soil microorganisms [27]. Biochar is suggested to improve soil quality, and there are reports of stimulated microbial response and loss of native SOC [28]. In addition, the effectiveness of biochar depends on its raw material and production conditions, quality and application rates, and type of soil [29]. However, whether the application of biochar in water-saving irrigation paddy fields manifests the same effect remains to be verified. With the development of rice production in China, rice water-saving irrigation technology has been widely applied to achieve high-efficiency, high-yield, energy-saving, and environmental protection. After the application of water-saving irrigation technology, the paddy field is in an unsaturated state in certain or most rice-growing stages, and the change of soil moisture is bound to affect the physical and chemical properties of soil. Accordingly, the transformation of biochar in the soil changes, but no relevant research is available yet. Studying the mechanism of biochar application on SOC and its components in water-saving irrigation paddy fields is important to cope with the shortage of water resources and reduction of SOC in water-saving irrigation paddy fields [30].

This study investigated the temporal and spatial distributions of SOC, water-soluble organic carbon (WSOC), and soil microbial biomass carbon (SMBC) in paddy fields under different irrigation methods and biochar application rates. The responses of catalase (CAT) and invertase (INV) to biochar application in paddy fields under water-saving irrigation were also analyzed. Apart from exploring the conjunct influences of rice water-saving irrigation technique and biochar application on SOC composition and related enzyme activities, this study also aimed to enrich the theory of water-saving irrigation and propose a reasonable water-carbon regulation model to provide a scientific basis for the sustainable utilization of soil and water resources in paddy fields.

## 2. Materials and Methods

### 2.1. Experimental Site

The experiment was conducted in 2017 at the State Key Laboratory of Hydrology-water Resources and Hydraulic Engineering of Hohai University, Kunshan Experiment Station (34°15′21″ N and 121°05′22″ E), located in the Eastern Taihu Lake region, China. The study area is part of the subtropical monsoon climate zone in Southeast China, with a mean annual air temperature, precipitation, evaporation, and sunshine duration of 15.5 °C, 1097.1 mm, 1375.9 mm, and 2104.9 h, respectively, and frost-free period of 232 days/year. The locals are accustomed to rice-wheat rotation. Soil in the experimental site is classified as dark-yellow hydromorphic paddy soil. The basic properties of this soil classification are as follows: organic matter of 21.71 g/kg for the top 0 to 18 cm layer, total N of 1.79 g/kg, total P of 1.4 g/kg, total K of 20.86 g/kg, pH of 7.4, and soil bulk density of 1.32 g/cm^3^ for the 0 to 30 cm layer.

### 2.2. Experimental Design

The experiment was laid out (pot size 80 cm × 50 cm × 70 cm) in a randomized block design with four treatments and three replicates. The four treatments were a combination of irrigation and rice straw biochar addition, and the two irrigation managements were controlled irrigation (CI) and flooding irrigation (FI). The three biochar managements under CI conditions were 0, 20, and 40 t/ha, and only one biochar application level (40 t/ha) was set under FI conditions. The four treatments were C0 (CI and 0 t/ha), C20 (CI and 20 t/ha), C40 (CI and 40 t/ha), and F40 (FI and 40 t/ha). The pots were individually constructed containers, which had a drainpipe buried at the bottom of the pot. The drainage can be manually controlled by the drainpipe. The soil substrate was taken from the local natural soil and was loaded and compacted layer-by-layer according to the order of soil sampling to ensure that the soil layer in the pots was consistent with the natural soil. All treatments were applied to the same pots during the experiment. The variety of rice planted in this experiment was Suxiangjing rice. Three to four seedlings per hill were transplanted with 13.0 × 25.0 cm hill spacing on 30 June 2017 and harvested on 31 October of the same year.

The rice straw biochar used in the experiment was provided by Zhejiang Biochar Engineering Technology Research Center. The biochar was spread in the pots manually and incorporated into soil (approximately 20 cm) using a shovel 1 day prior to transplantation of rice in 2016. The main properties of biochar were as follows [31]: C content of 42.6%, total N of 0.75%, total P of 0.15%, total K of 1.06%, special surface area of 81.9 m^2^/g, total pore volume of 0.08 cm^3^/g, and pH of 10.1. For the FI treatment, a standing water depth of 3 to 5 cm was maintained after transplantation, except for the late tillering and yellow maturity stages. For the CI treatment, the ponded water depth was only kept at 10 to 30 mm in the regreening stage. Irrigation was applied to maintain soil moisture, and standing water was avoided in other stages, except during periods of pesticide and fertilizer applications, with a combination of soil moisture for root layer accounting for 60% to 80% of saturated soil moisture content as an irrigation control indicator [32]. A conventional fertilizer application was managed in accordance with local farmers’ customary fertilization methods and fertilization amount, such as 328.48, 45.00, and 63.75 kg/hm^2^ of N, P, and K fertilizers, respectively.

### 2.3. Soil Sampling and Analysis

Rice-wheat rotation was carried out in the Taihu Lake region in China. Starting from the end of June when rice was transplanted and ending in October when rice was harvested, the rice growth period lasted for approximately 125 days. Soil samples were collected four times before transplantation (27 June), at the tillering (30 July), jointing and booting (28 August), and milk stages (29 September). Soil samples of 0 to 10, 10 to 20, and 20 to 40 cm were collected, using the “S-shape” method in each pot. After the visible plant residues and gravels were removed, the soil samples were divided into two parts as follows: one was fresh soil samples stored in a refrigerator at 4 °C to determine SMBC and WSOC, and the other was naturally air-dried and passed through 20- and 100-mesh sieves to determine soil enzyme activity and total SOC.

Specific determination methods: SOC was determined by K dichromate oxidation [33]. WSOC was determined using the K dichromate-concentrated sulfuric acid external heat capacity method (wet oxidation method) [34]. In a study by Wu et al. [35], SMBC was determined using the fumigation extraction method of chloroform. The soil INV activity was determined by 3,5-dinitrosalicylic acid colorimetry. Soil CAT activity was determined by K permanganate titration. The water layer was recorded at 8:00 by using vertical rulers, which were pre-embedded in the pot. Soil moisture was measured with a portable time domain reflectometer when no water layer existed. A 1 L measuring cup was used for irrigation, and the amount of irrigation was recorded in time.

### 2.4. Statistical Analysis

Excel 2010 was used to initially analyze data and create a database for drawing related charts. Statistical analyses were performed using standard procedures for a randomized plot design (SPSS 22.0, SPSS Inc., Chicago, IL, USA). Significance was calculated on the basis of F-tests and least significant differences at 0.05 probability level. Data principal component analysis (PCA) and mapping were completed by using Origin 2017.

## 3. Results

### 3.1. Changes in Total SOC Content

The change trend of total SOC content in different growth stages of rice was relatively consistent (Table 1). During the regreening period, the total SOC of different soil layers accumulated and continuously increased, reached its peak at the tillering stage, and decomposed and continuously decreased at the jointing and booting and milk stages. The fluctuation of total organic carbon content in the paddy soil with season indicated that an active carbon component conversion existed in the soil. The total SOC content of CI during the entire growth period was lower than that of conventional irrigation, and significant differences between 10 cm and 40 cm soil layer were observed at the tillering stage, 20 cm and 40 cm at the jointing and booting stage, and 0 cm and 40 cm at the milk stage (*p* < 0.05). The SOC contents in 0 to 10, 10 to 20, and 20 to 40 cm soil under conventional irrigation were 0.80, 0.78, and 1.32 g/kg higher than those under CI, respectively. In addition, the SOC content in the controlled irrigated paddy fields decreased by 5.7% to 13.3%. The total SOC content in paddy fields under different water-carbon regulation scenarios gradually decreased with the deepening of soil layer. The SOC contents of C0, C20, and C40 treatments, in the 0 to 10 cm soil layer, were 10.16, 11.77, and 13.22 g/kg, respectively. These values were 7.73%, 12.39%, and 9.32% and 24.61%, 33.19%, and 34.53% lower than those in the 10 to 20 and 20 to 40 cm soil layers, respectively. The SOC content of paddy fields increased with biochar application. In the 0 to 40 cm soil layer, the total organic carbon content treated with high (C40) and medium-low (C20) level biochar was 19.75% and 11.60% higher than that of soil treated with no biochar (C0). The SOC content remarkably increased by 4.0% to 26.7% with biochar application due to the direct increase of SOC by the external organic matter input. The biochar application affected the input of total organic carbon in the soil. Irrigation affected the conversion of SOC. Consequently, the change of total SOC content was determined. A two-way ANOVA was conducted to determine the changes in SOC content in different water-carbon regulation treatments of rice growth stages. The results showed that biochar application presented a significant (*p* < 0.05) and highly significant difference (*p* < 0.01) in the jointing and booting and milk stages of rice, respectively. A significant (*p* < 0.05) effect of irrigation was also observed on the change of SOC content during the milk stage.

### 3.2. Changes in Active SOC Content

The WSOC content in paddy fields fluctuated in the range of 166.57 to 403.86 mg/kg under different water-carbon regulations (Table 2). Such content in all treatments continuously increased at the tillering stage, reached the highest value at the jointing and booting stage, and remarkably decreased at the milk stage. The WSOC content in the rice field decreased with soil depth. Evidently, the WSOC content of C0, C20, and C40 treatments in the 0 to 10 soil layer was 7.97% to 16.05% and 25.09% to 26.44% higher than those in the 10 to 20 and 20 to 40 cm soil layers, respectively. The WSOC content of the rice field increased with biochar application, and that with high biochar treatment was higher than that with low biochar treatment, increasing the WSOC content by 4.0% to 52.4%. The results showed that the WSOC content of conventional irrigation treatment was lower than that of CI treatment at the same level of biochar application. The WSOC contents of conventional irrigation treatment (F40) were 43.28, 52.07, and 58.5 mg/kg lower than those of CI treatment (C40) in the 0 to 10, 10 to 20, and 20 to 40 cm soil layers, respectively, and the difference was significant (*p* < 0.05). The application of water-saving irrigation increased the WSOC content of rice field by 13.8% to 26.1%. This increase could be due to the alternation of wetting and drying in paddy soil under CI conditions, which was beneficial to microbial reproduction and decomposition of soil organic matter to produce substantial water-soluble carbon. The combination of water-saving irrigation and high biochar application improved the WSOC content in paddy soil. The biochar application highly significantly affected the WSOC content at all stages of rice growth (*p* < 0.01). By contrast, irrigation highly significantly affected the tillering and milk stages (*p* < 0.01) and significantly affected the jointing and booting stage (*p* < 0.05).

The SMBC content fluctuated within the range of 83.36 to 168.65 mg/kg during the entire growth period (Table 3). The variation of SMBC under different water and carbon managements was consistent with that of WSOC, which showed an increasing and then decreasing trend and reached the maximum value during the entire growth period at the jointing and booting stage. The SMBC content in CI increased by 0.88% to 11.11% as compared with conventional irrigation. The differences of the 0 to 10, 10 to 20, and 20 to 40 cm soil layers during the milk period were significant (*p* < 0.05). CI was conducive to microbial accumulation. The SMBC content decreased with the increase of soil depth. The contents of C0, C20, and C40 in the 10 to 20 cm soil layer decreased by 14.37%, 17.87%, and 17.12% and 23.65%, 32.74%, and 24.51% as compared with those in the 0 to 10 and 20 to 40 cm soil layers, respectively. Under the same irrigation mode, the SMBC content increased with the biochar level and increased by 7.0% to 40.8% when biochar was applied as compared with that without biochar application. This result indicated that the application of high biochar was beneficial to the improvement of microbial carbon content in soils in combination with CI. The results of two-way ANOVA showed that the application of biochar highly significantly affected the SMBC content during the entire growth period of rice (*p* < 0.05).

### 3.3. Changes in Soil Enzyme Activity

The soil CAT activity of each treatment in different growth stages showed a vertical distribution pattern of increasing first and then decreasing (Table 4). Biochar application improved the soil CAT activity in the same depth of water-saving irrigation paddy field, and the effect increased with biochar application. The soil CAT activity treated with C40 and C20 in the 20 to 40 cm soil layer was 5.47% to 8.89% and 1.29% to 5.47% higher than that of C0 treatment (*p* < 0.05). Such activity at the same depth decreased with the extension of the growth period. CI improved the CAT activity in soils as compared with the conventional one. For the entire research depth (0 to 40 cm), the soil CAT contents of C40 were 4.26%, 5.16%, and 12.44% higher than those of F40 at the tillering, jointing and booting, and milk stages, respectively, under the same biochar amount (*p* < 0.05). The results showed that biochar application and irrigation highly significantly affected the soil CAT activity at the jointing and booting and milk stages (*p* < 0.01). Biochar application at the tillering stage significantly affected such activity (*p* < 0.05).

The vertical distribution of INV activity in paddy soils was basically the same at different growth stages (Table 5) and linearly decreased with the increase of soil depth. The soil INV activity under CI was generally shown as C40 > C20 > C0, and that of C40 and C20 treatments was 3.21% to 23.38% and 35.26% to 73.43% higher than that of C0 treatment at all growth stages. Such activity in the same soil depth at different growth stages was lower at the FI condition. With regard to the entire research depth (0 to 40 cm), the INV contents in the soil treated with C40 were 4.88%, 9.90%, and 20.98% higher than those treated with F40 at the tillering, jointing and booting, and milk stages, respectively.

In the 0 to 10 cm soil layer, the soil INV activity first decreased and then increased with the growth period, and the differences between C0 and C40, and C20 and C40 treatments were significant (*p* < 0.05). In the 10 to 20 cm soil layer, the soil INV activity gradually decreased with rice growth. Significant differences were observed among C0, C20, and C40 treatments at the tillering and milk stages and between C20 and C40 at the jointing and booting stage (*p* < 0.05). In the 20 to 40 cm soil layer, the soil INV activity showed an increasing trend with the prolongation of growth period, and the C0, C20, and C40 treatments were significantly different at the milk stage (*p* < 0.05). The biochar application at the tillering stage significantly affected the soil INV activity (*p* < 0.05), especially after the tillering stage (*p* < 0.01).

### 3.4. Correlation Analysis of Various Indicators

**Figure 1:**
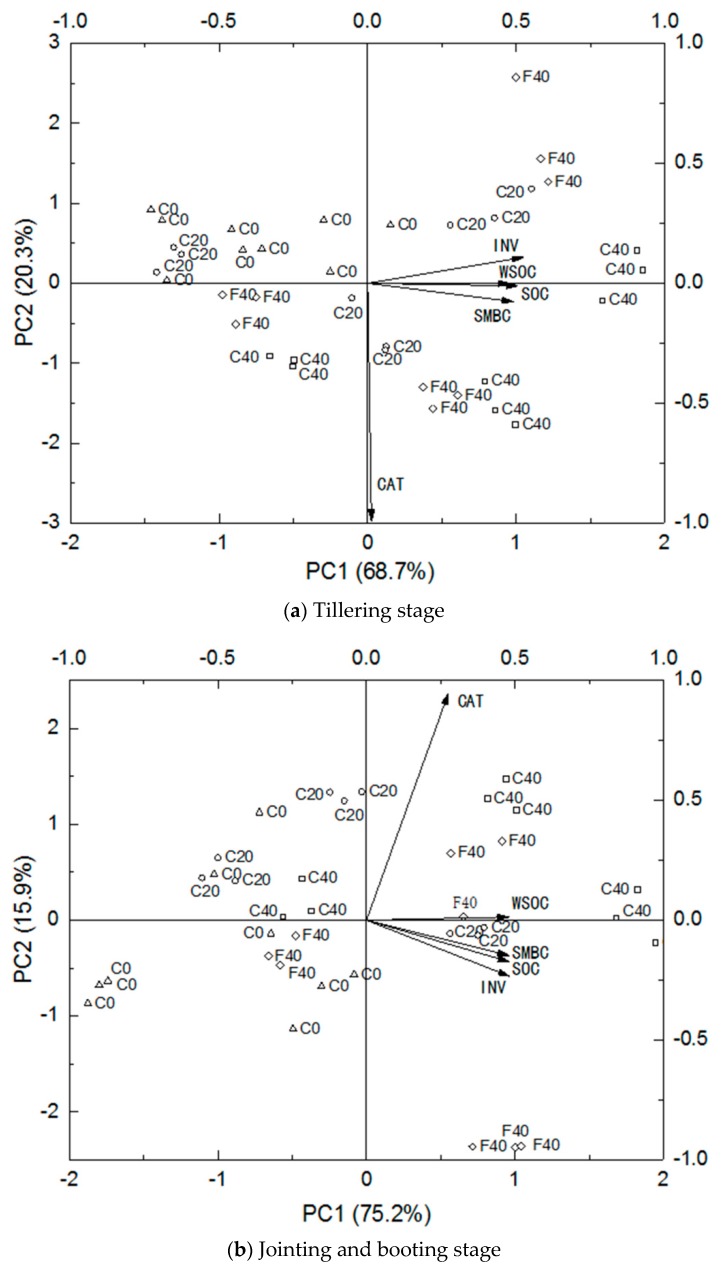

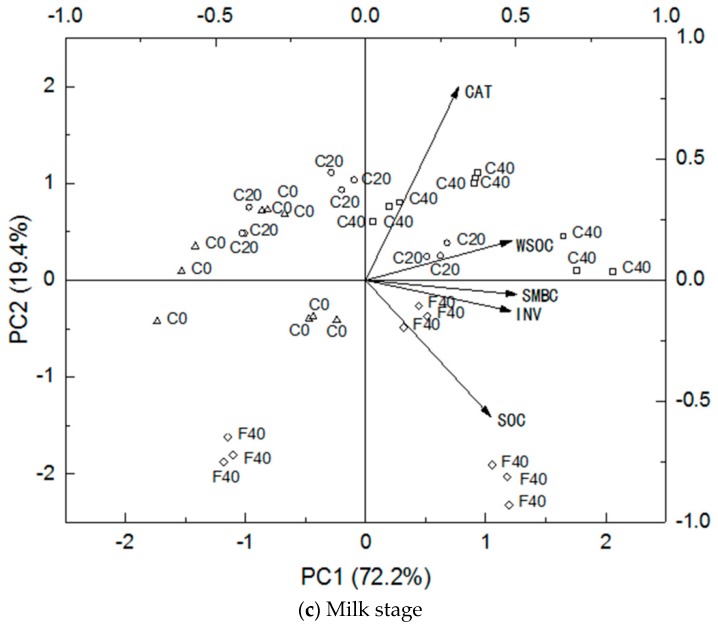
Principal component analysis (PCA) of the relationships of the fractions of SOC and its components with soil enzyme activities at different growth stages of rice. (**a**): PCA biplot at tillering stage; (**b**): PCA biplot at jointing and booting stage; (**c**): PCA biplot at milk stage.

PCA was conducted at the tillering, jointing and booting, and milk stages of rice on the basis of the SOC data and its components and related enzymes in all treatments (Figure 1). The first two principal components with eigenvalues greater than one were systematically extracted. At the tillering stage (a), the two principal component eigenvectors were 3.43 and 1.01. PC1 and PC2 jointly explained the 89.00% variation, and the contribution rate of the first principal component was 68.68%. The loading analysis of principal component eigenvalues showed that high positive loads in the first principal component represented INV activity, SOC, WSOC, and SMBC. This result indicated that the first principal component mainly reflected the catalytic characteristics of soil INV. The high negative load of the second principal component represented the CAT activity. The second principal component mainly reflected the catalytic properties of soil CAT. The F40 and C40, and C0 and C20 treatments scored high on the PC1 and PC2 axes, respectively. This result indicated that the application of high-volume biochar treatment greatly contributed to the soil INV catalytic process. Meanwhile, CI and medium-volume biochar treatment greatly contributed to the soil CAT catalytic process.

At the jointing and booting stage (b), the variance percentage of PC1 that explained the original variables was 75.24%, and that of PC2 was 15.87%. The cumulative variance of the two principal components was 91.12%. After extracting loads greater than 0.45 from the factor load matrix, the INV activity, SOC, WSOC, and SMBC were the major positive loads in the first principal component, and CAT activity was the largest positive load in the second principal component. Therefore, PC1 and PC2 should represent the SOC conversion process associated with soil INV and SOC conversion process related to soil CAT, respectively. The principal component score matrix implied that the high scores on the PC1 axis were F40 and C40 treatments. The high scores on the PC2 axis were C20 and C40 treatments, and the C40 treatment exhibited high scores on both axes. This result suggested that CI and high-volume biochar addition demonstrated a considerable contribution to the SOC transformation related to soil INV and CAT at the jointing and booting stage.

At the milk stage (c), the two principal components explained that 91.64% of the total variance of the original variables and the extraction of principal components were ideal. Similar to the tillering and jointing and booting stages, the high positive loads on the PC1 axis represented SMBC, WSOC, and INV activity. Meanwhile, the CAT on the PC2 axis showed high positive loads, and SOC presented high negative ones. PC1 and PC2 represented the active SOC synthesis process related to soil INV and SOC decomposition process related to soil CAT, respectively. The comprehensive scores of the biplot (Figure 1c) showed that C40 and F40 and C20 and C40 scored high on PC1 and PC2, respectively. Thus, the application of high-volume biochar greatly contributed to soil INV activity and SMBC and WSOC contents, whereas CI and biochar addition made important contributions to soil CAT activity and SOC content.

The correlation analysis (Table 6) suggested that SOC, WSOC, and SMBC were highly significantly correlated in rice paddy fields at different growth stages (*p* < 0.01). This notion indicated that different forms of organic carbon in the soil accompanied by rice growth and development were closely related to one another. The Pearson correlation coefficients of soil CAT activity and SOC, WSOC, and SMBC in the tillering stages were 0.039, 0.025, and 0.087, respectively, and no significant correlation was observed. This result showed that soil CAT exhibited minimal influence on the formation and transformation of SOC and active components in this growth stage. At the jointing and booting stage, the soil CAT activity was significantly correlated with SOC and SMBC (*p* < 0.05) and highly significantly correlated with WSOC (*p* < 0.01). After the milk period, soil CAT activity was highly significantly correlated with WSOC and SMBC in addition to SOC, indicating that soil CAT was gradually involved in the metabolism and transformation of organic carbon after tillering, and its activity could characterize the conversion rate of soil organic matter. Soil INV activity demonstrated a positive significant correlation with SOC, WSOC, and SMBC during the entire growth period of rice (*p* < 0.01). Therefore, the soil INV activity was closely associated with the total SOC content and its components.

## 4. Discussion

### 4.1. Effects of Biochar Application on SOC and Its Active Components in Paddy Fields under Water-Saving Irrigation

The application of biochar can rapidly enhance the soil carbon pool. Laird et al. [24] found that the SOC content increased with the addition of biochar under the same fertilization conditions. Ma et al. [36] found that biochar could remarkably increase the SOC, WSOC, and SMBC content of gray desert soil through pot experiments in Xinjiang, China. In addition, the higher the pyrolysis temperature of biochar and the larger the application amount, the more evident the improvement of SOC content. Several studies [37,38] emphasized that the stable structure inside the biochar inhibited the surface oxidation of organic carbon, increased the stability of SOC against microbial degradation, and reduced the mineralization rate of SOC, thereby improving the SOC content. Durenkamp et al. [39] found that microbial biomass carbon in clay soil increased with the addition of biochar. The active organic carbon content in biochar directly affected the organic carbon composition in soils. In this experiment, biochar application could remarkably increase the SOC content and its active components. The effect increased with biochar application, which was consistent with the conclusions of previous research [24,27,36]. This result might be attributed to the improved crop biomass, especially root biomass, after biochar application, which increased the input of fresh organic carbon. Moreover, the contents of soil WSOC and SMBC increased with biochar application. Biochar application was beneficial to the accumulation of SOC and the improvement of soil properties and fertility. Several studies have shown that soil moisture is the key driving factor in the carbon cycle process. Within a certain range of changes, soil moisture manifested a significant correlation with the transformation of organic carbon [40,41]. The remarkable changes in soil moisture content and its distribution caused by the change in irrigation mode were bound to have an important impact on SOC and its active components. This study found that water-saving irrigation reduced the SOC content in paddy soils but increased soil WSOC and SMBC contents. Water-saving irrigation promoted SOC decomposition. The result of the combined application of biochar and water-saving irrigation in this experiment indicated that the application of biochar would also increase SOC and its components in the paddy fields under water-saving irrigation. This phenomenon was due to the promotional effect of biochar application. By contrast, water-saving irrigation changed the soil water environment in the field. No water layer was established in water-saving irrigation paddy fields after the regreening stage of rice. The plough layer was effectively aerated, and the hydrothermal conditions were suitable; that is, these conditions were conducive to the mineralization and decomposition of organic matter and improvement of soil microbial activity. A direct correlation was observed between soil microbial activity and INV and CAT activities [42]. The correlation analysis results showed that CAT gradually participated in the synthesis, decomposition, and transformation of organic carbon after the tillering stage of rice. Meanwhile, INV was closely related to carbon transformation during the entire growth period. The soil conditions could possibly improve the quantity and activity of the related enzymes after the combination of biochar and water-saving irrigation.

### 4.2. Effects of Biochar Application on Soil Enzyme Activity in Paddy Fields under Water-Saving Irrigation

As catalysts for chemical reactions, soil enzymes were closely related to the decomposition rate of SOC and turnover pattern of the SOC pool [43]. A previous study suggested that biochar can remarkably improve the soil CAT activity and soil enzyme index but showed no remarkable effect on the INV activity [44]. However, certain studies also found that biochar application remarkably increased the activities of soil urease, alkaline phosphatase, and INV, and the combination of biochar and N fertilizer remarkably increased the activity of INV as compared with single N application [44,45]. This study showed that biochar application played an important role in promoting the activities of CAT and INV in water-saving irrigated paddy soil, and the effect increased with biochar application. This occurrence was partly due to the increased contents of soil and dissolved organic matter after biochar addition, which were conducive to improving the composition and abundance of soil bio-community and enzymatic activity. Biochar presented a large comparative surface area and pore distribution [46], which could promote the formation of soil aggregates, improve soil aeration and water retention capacity, and positively affect the soil microbial metabolism [47]. In this study, the effect of biochar on soil CAT activity was weaker than that on INV activity, which could be related to the soil’s physical and chemical properties and crop types. Many studies have shown that soil enzyme activity is related to water condition. Gao [48] showed that water could remarkably affect soil peroxidase activity through indoor incubation experiments, and such activity was stronger under flooding conditions. Wan et al. [49] found that the activities of acid phosphatase, INV, and urease decreased with the increase of soil moisture, but the activities of CAT increased with the increase of soil moisture. Wetting–drying cycles increased the activities of acid phosphatase, INV, urease, and CAT. This study showed that CI could improve the activities of soil CAT and INV, which was consistent with the results of Xiao et al. [50]. The soil INV activity under CI (C40) gradually increased with the extension of growth period. By contrast, the soil INV activity reduced along with the growth period under conventional irrigation (F40). This outcome could be due to the slight deficit of soil moisture caused by CI. The alternation of wetting–drying improved soil aeration and accelerated the reproduction cycle of soil microorganisms, and considerable enzymes were secreted by soil microbial biomass with the growth of rice. However, excessive water in soils decreased soil permeability and inhibited soil enzyme activity under conventional irrigation.

## 5. Conclusions

Biochar application increased the organic carbon content by 4.0% to 26.7% in paddy fields under water-saving irrigation, and the contents of soil WSOC and SMBC increased by 4.0% to 52.4% and 7.0% to 40.8%, indicating that the joint regulation of water-saving irrigation and biochar was conducive to SOC accumulation and improvement of soil properties and fertility. The recommended biochar application rate was 40 t/ha. The correlations of total SOC, soil WSOC, and SMBC were highly significant in the entire growth period of rice. This result showed that an active conversion of carbon components was observed in the soil with rice growth. Under the combined utilization of water-saving irrigation and biochar, soil CAT activity increased by 1.8% to 8.7%, and soil INV activity increased by 3.2% to 74.1%. Biochar application could improve the soil microbial activity and nutrient conversion ability of water-saving irrigation paddy fields. Soil CAT was significantly correlated with SOC and its active components at the jointing and booting and milk stages (*p* < 0.05). Soil INV was significantly associated with SOC and its active components during the entire growth period (*p* < 0.01). Soil CAT and INV were closely related to the mineralization, decomposition, and transformation of soil organic matter.

## Figures and Tables

**Table 1:** Characteristics of total soil organic carbon (SOC) under different water-carbon regulation scenarios in paddy fields (g/kg).

Soil Depth	Treatments	Before Transplantation	Tillering Stage	Jointing and Booting Stage	Milk Stage
0–10	C0	10.12b	10.58c	10.33c	9.56d
	C20	10.55b	12.64b	12.30b	10.36c
	C40	12.07a	14.00a	13.63a	12.03b
	F40	12.15a	14.12a	13.96a	13.97a
10–20	C0	9.08c	10.03d	9.84c	8.25d
	C20	9.64b	11.81	10.10b	9.02c
	C40	11.07a	12.68b	12.43a	10.86b
	F40	11.22a	13.74a	12.97a	11.60a
20–40	C0	7.76b	8.67b	7.24c	7.06d
	C20	7.71b	7.48c	8.51b	7.60c
	C40	9.02a	8.97b	8.38b	8.62b
	F40	9.36a	9.77a	9.84a	10.34a
**Two-way ANOVA**					
Biochar application		0.013 *	0.096	0.042 *	0.006 **
Irrigation		0.748	0.492	0.387	0.031 *

Note: Different letters, such as a and b, within a column in the same soil depth indicate significant differences (*p* < 0.05). The results of two-way variance analysis are shown as *p*-values. * and ** indicate that the effect of biochar application or irrigation on a certain index is significant at *p* < 0.05 and *p* < 0.01, respectively.

**Table 2:** Characteristics of soil water-soluble organic carbon (WSOC) under different water-carbon regulation scenarios in paddy fields (mg/kg).

Soil Depth	Treatments	Before Transplantation	Tillering Stage	Jointing and Booting Stage	Milk Stage
0–10	C0	208.39ab	221.50c	305.76c	245.78c
	C20	205.10b	259.77b	364.24b	301.05b
	C40	218.01a	303.63a	403.86a	365.58a
	F40	201.39b	263.61b	369.31b	310.30b
10–20	C0	177.60a	206.58c	259.63c	199.94c
	C20	175.55a	251.48b	320.91b	284.36b
	C40	185.19a	280.39a	368.34a	326.44a
	F40	184.14a	206.85c	338.45b	273.64b
20–40	C0	164.63b	190.16b	228.89c	198.96c
	C20	166.12b	169.94c	308.71b	259.01b
	C40	169.33b	225.32a	332.96a	290.42a
	F40	176.11a	166.57c	295.23b	211.41c
**Two-way ANOVA**					
Biochar application		0.573	0.002 **	0.000 **	0.000 **
Irrigation		0.679	0.002 **	0.029 *	0.000 **

Note: Different letters, such as a and b, within a column in the same soil depth indicate significant differences (*p* < 0.05). The results of two-way variance analysis are shown as *p*-values. * and ** indicate that the effect of biochar application or irrigation on a certain index is significant at *p* < 0.05 and *p* < 0.01, respectively.

**Table 3:** Characteristics of soil microbial biomass carbon (SMBC) under different water-carbon regulation scenarios in paddy fields (mg/kg).

Soil Depth	Treatments	Before Transplantation	Tillering Stage	Jointing and Booting Stage	Milk Stage
0–10	C0	102.12b	117.78c	121.41c	105.40c
	C20	111.13a	129.05bc	143.82b	140.32b
	C40	113.06a	149.01a	168.65a	161.60a
	F40	109.54a	135.57ab	147.80b	142.63b
10–20	C0	89.37c	91.59c	104.25c	99.24d
	C20	99.43b	106.61b	124.05b	108.69c
	C40	103.60b	129.84a	133.67ab	133.71a
	F40	110.29a	127.10a	146.43a	120.18b
20–40	C0	86.35a	83.36c	92.37b	87.36bc
	C20	86.83a	100.44b	96.47b	81.00c
	C40	90.61a	119.58a	126.47a	115.73a
	F40	89.03a	109.42b	123.78a	94.19b
**Two-way ANOVA**					
Biochar application		0.120	0.000 **	0.001 **	0.001 **
Irrigation		0.911	0.205	0.676	0.067

Note: Different letters, such as a and b, within a column in the same soil depth indicate significant differences (*p* < 0.05). The results of two-way variance analysis are shown as *p*-values, and ** indicates that the effect of biochar application or irrigation on a certain index is significant at the 0.01 level.

**Table 4:** Characteristics of soil catalase (CAT) activity under different water-carbon regulation scenarios in paddy fields (mg/L).

Soil Depth	Treatments	Tillering Stage	Jointing and Booting Stage	Milk Stage
0–10	C0	8.45a	8.38d	8.15c
	C20	8.32a	8.75c	8.69b
	C40	8.67a	9.04b	8.97a
	F40	7.90b	8.12a	7.94d
10–20	C0	8.49c	8.71c	8.65c
	C20	8.99b	9.03ab	8.81b
	C40	9.40a	9.25a	9.21a
	F40	9.35a	8.98b	8.55c
20–40	C0	8.44c	8.12c	7.89c
	C20	8.55c	8.59a	8.26b
	C40	9.09a	8.59a	8.66a
	F40	8.81b	8.45b	7.39d
**Two-way ANOVA**				
Biochar application		0.011 *	0.001 **	0.001 **
Irrigation		0.064	0.004 **	0.000 **

Note: Different letters, such as a and b, within a column in the same soil depth indicate significant differences (*p* < 0.05). The results of the two-way variance analysis are shown as *p*-values. * and ** indicate that the effect of biochar application or irrigation on a certain index is significant at *p* < 0.05 and *p* < 0.01, respectively.

**Table 5:** Characteristics of soil invertase (INV) activity under different water-carbon regulation scenarios in paddy fields (mg/g).

Soil Depth	Treatments	Tillering Stage	Jointing and Booting Stage	Milk Stage
0–10	C0	0.187c	0.186c	0.187b
	C20	0.248b	0.201c	0.203b
	C40	0.284a	0.269a	0.304a
	F40	0.248b	0.238b	0.284a
10–20	C0	0.155c	0.158c	0.156b
	C20	0.185b	0.153c	0.135c
	C40	0.226a	0.218a	0.211a
	F40	0.217a	0.191b	0.226a
20–40	C0	0.119c	0.123b	0.085d
	C20	0.138ab	0.128b	0.124b
	C40	0.134bc	0.147a	0.230a
	F40	0.150a	0.146a	0.104c
**Two-way ANOVA**				
Biochar application		0.041 *	0.010 **	0.001 **
Irrigation		0.678	0.304	0.11

Note: Different letters, such as a and b, within a column in the same soil depth indicate significant differences (*p* < 0.05). The results of two-way variance analysis are shown as *p*-values. * and ** indicate that the effect of biochar application or irrigation on a certain index is significant at *p* < 0.05 and *p* < 0.01, respectively.

**Table 6:** Pearson correlation coefficients of SOC and its components with soil enzyme activities at different growth stages of rice.

Growth Period		Tillering Stage					Jointing and Booting Stage					Milk Stage				
Indicator		SOC	WSOC	SMBC	CAT	INV	SOC	WSOC	SMBC	CAT	INV	SOC	WSOC	SMBC	CAT	INV
Tillering stage	SOC	1														
	WSOC	0.757 **	1													
	SMBC	0.784 **	0.720 **	1												
	CAT	0.039	0.025	0.087	1											
	INV	0.920 **	0.816 **	0.863 **	-	1										
Jointing and booting stage	SOC	0.942 **	0.699 **	0.827 **	-	-	1									
	WSOC	0.736 **	0.775 **	0.911 **	-	-	0.802 **	1								
	SMBC	0.845 **	0.711 **	0.907 **	-	-	0.836 **	0.859 **	1							
	CAT	-	-	-	0.691 **	-	0.382 *	0.481 **	0.382 *	1						
	INV	-	-	-	-	0.943 **	0.910 **	0.821 **	0.842 **	-	1					
Milk stage	SOC	0.868 **	0.584 **	0.827 **	-	-	0.921 **	0.755 **	0.830 **	-	-	1				
	WSOC	0.675 **	0.846 **	0.847 **	-	-	0.702 **	0.936 **	0.765 **	-	-	0.628 **	1			
	SMBC	0.860 **	0.895 **	0.879 **	-	-	0.851 **	0.869 **	0.879 **	-	-	0.782 **	0.853 **	1		
	CAT	-	-	-	0.456 **	-	-	-	-	0.820 **	-	0.070	0.626 **	0.517 **	1	
	INV	-	-	-	-	0.765 **	-	-	-	-	0.847 **	0.754 **	0.791 **	0.859 **	-	1

Note: * and ** indicate significant correlation at the 0.05 and 0.01 levels, respectively.
